# Structure of the HHARI Catalytic Domain Shows Glimpses of a HECT E3 Ligase

**DOI:** 10.1371/journal.pone.0074047

**Published:** 2013-08-15

**Authors:** Donald E. Spratt, Pascal Mercier, Gary S. Shaw

**Affiliations:** Department of Biochemistry, Schulich School of Medicine and Dentistry, University of Western Ontario, London, Ontario, Canada; University of Saskatchewan, Canada

## Abstract

The ubiquitin-signaling pathway utilizes E1 activating, E2 conjugating, and E3 ligase enzymes to sequentially transfer the small modifier protein ubiquitin to a substrate protein. During the last step of this cascade different types of E3 ligases either act as scaffolds to recruit an E2 enzyme and substrate (RING), or form an ubiquitin-thioester intermediate prior to transferring ubiquitin to a substrate (HECT). The RING-inBetweenRING-RING (RBR) proteins constitute a unique group of E3 ubiquitin ligases that includes the Human Homologue of *Drosophila* Ariadne (HHARI). These E3 ligases are proposed to use a hybrid RING/HECT mechanism whereby the enzyme uses facets of both the RING and HECT enzymes to transfer ubiquitin to a substrate. We now present the solution structure of the HHARI RING2 domain, the key portion of this E3 ligase required for the RING/HECT hybrid mechanism. The structure shows the domain possesses two Zn^2+^-binding sites and a single exposed cysteine used for ubiquitin catalysis. A structural comparison of the RING2 domain with the HECT E3 ligase NEDD4 reveals a near mirror image of the cysteine and histidine residues in the catalytic site. Further, a tandem pair of aromatic residues exists near the C-terminus of the HHARI RING2 domain that is conserved in other RBR E3 ligases. One of these aromatic residues is remotely located from the catalytic site that is reminiscent of the location found in HECT E3 enzymes where it is used for ubiquitin catalysis. These observations provide an initial structural rationale for the RING/HECT hybrid mechanism for ubiquitination used by the RBR E3 ligases.

## Introduction

The ubiquitin-signaling pathway plays a vital role in intracellular signaling and protein turnover in the cell. This pathway involves a cascade of enzymes (E1 ubiquitin-activating, E2 ubiquitin-conjugating, and E3 ubiquitin ligase) to sequentially transfer an ubiquitin moiety to a target protein by forming an isopeptide bond between the C-terminus of ubiquitin and an ε-amide of a lysine on the substrate protein or growing ubiquitin chain [1]. The E3 ligases, which are responsible for recognizing the substrate protein and ubiquitin attachment specificity, have generally been classified under two different categories depending upon their structure and mechanism. For instance, the Really Interesting New Gene (RING) and U-box E3 ligases act as scaffolds to properly orient an E2 ~ ubiquitin thioester complex to transfer its ubiquitin cargo to a substrate [2,3]. In contrast, the Homologous to E6AP Carboxyl Terminus (HECT) E3 ligases play a more direct role in ubiquitin transfer by forming a catalytic thioester intermediate with the C-terminus of ubiquitin before it is transferred to a substrate [4,5].

The RING-inBetweenRING-RING (RBR) E3 ubiquitin ligases [6,7] are a unique group of E3 ligases that include two putative RING domains separated by an inBetweenRING (IBR) domain (ie. RING1-IBR-RING2) near their C-termini. Originally these enzymes were thought to function in a similar manner as the RING E3 ligases with either the RING1 or RING2 domain acting as an adaptor to facilitate ubiquitin transfer from the E2 enzyme to a substrate. More recently, it has been shown that several RBR E3 ligases including HHARI, parkin, and heme-oxidized-IRP2 ubiquitin ligase 1 interacting protein (HOIP) use a unique hybrid mechanism combining aspects from both the RING and HECT E3 ligases [8–10]. In this hybrid mechanism, the RING1 domain is proposed to recruit the E2 enzyme UbcH7 and facilitate the transthiolation of ubiquitin to a conserved cysteine within the RING2 domain, prior to ubiquitin off-loading to a substrate protein [8–11].

Human homolog of *Drosophila* Ariadne-1 (HHARI/ARI1) is a member of the RBR E3 ligase family involved in the ubiquitylation of substrate proteins including single-minded 2 (SIM2) [12] and translation initiation factor 4E homologous protein (4EHP) [13]. Recent studies have shown that HHARI is highly expressed in the nucleus and promotes cellular proliferation [14] and is susceptible to oxidative damage leading to HHARI insolubility [15]. The gene homolog in *Drosophila* , *Ariadne-1*, is expressed in all tissues during development. Null alleles markedly shorten life expectancy and substitutions of conserved cysteines throughout the RBR sequence are lethal [16]. HHARI has been shown to interact with the E2 conjugating enzymes UbcH7, UbcH8, UbcM4 and UbcD10 in human, mouse and fly [16–19]. It has also been demonstrated that the RING1 and IBR domains of HHARI are required for E2 recruitment [16,18,19] and that modifying the linker between the RING1 and IBR domains or substituting the RING1 domain of HHARI with its RING/RING1 cognate from c-Cbl or parkin abolishes HHARI’s ability to interact with UbcH7 [18].

In this work we have determined the three-dimensional solution structure of the catalytic RING2 domain from HHARI. We show that HHARI RING2 forms a compact structure that features two bound zinc ions that does not resemble a typical RING E3 ligase fold. A key observation to the RING2 fold is the presence of aromatic residues that maintain the protein structure and are present in all RING2 domains for the RBR E3 ligase family. Further we show that an exposed loop, carrying the catalytic cysteine and an adjacent histidine residue, is poised to accept and transfer ubiquitin reminiscent of a similar region found in HECT E3 ligases.

## Materials and Methods

### Protein expression and purification

The RING2 domain of human HHARI (residues 325-396) with a C357S substitution was synthesized by DNA 2.0 (Menlo Park, CA, USA) and cloned into a modified pGEX-6P-2 vector having an *N-*terminal GST tag followed by a TEV cleavage site (ENLYFQ), as previously described [11]. The GST-TEV-HHARI RING2 construct was transformed into *Escherichia coli* BL21(DE3)-RIL (Stratagene) and grown at 37°C in M9 media supplemented with ^15^NH_4_Cl (1 g/L), ^13^C_6_-glucose (2 g/L), 100 µg/mL ampicillin and 34 µg/mL chloramphenicol. When the culture OD_600_ reached 0.6, the media was supplemented with 500 µM ZnCl_2_ and the temperature was dropped to 16°C. Once the cells reached an OD_600_ of 0.8, the cultures were induced with 1 mM IPTG at 16°C for 20 hours. Cells were harvested by centrifugation at 6000 x g for 10 minutes at 4°C.

Cell pellets were re-suspended in 20 mL wash buffer (20 mM Tris-HCl, 120 mM NaCl, 5 mM DTT, pH 7.4) with 1 mM PMSF and an EDTA-free protease inhibitor tablet (Roche, Mississauga, Ontario), lysed using an EmulsiFlex-C5 homogenizer (Avestin, Ottawa, Ontario), and clarified by centrifugation (38,000 rpm for 1 hr at 4°C). The supernatant was then filtered (0.45 m, Millipore, Mississauga, ON, Canada) and loaded onto a 5µ mL GSTrap FF column (GE Healthcare) pre-equilibrated with wash buffer at a flow rate of 0.5 mL/min using an AKTA FPLC (GE Healthcare). After the column was washed with 20 column volumes of wash buffer at 3 mL/min, the protein was eluted with elution buffer (20 mM Tris-HCl, 120 mM NaCl, 10 mM glutathione, pH 7.4) at a flow rate of 2 mL/min. Fractions containing eluted protein were pooled and TEV protease was added to cleave the GST tag (1 mg TEV/50 mg protein) for 1 hour at 25°C. The TEV-cleaved protein was dialysed against 20 mM Tris-HCl, 120 mM NaCl, pH 8.5 at 25°C, followed by dialysis against wash buffer overnight at 4°C. The protein solution was then loaded onto a 5 mL GSTrap FF column at a flow rate of 0.5 mL/min and the flowthrough containing the HHARI RING2 domain was pooled. The protein was concentrated and loaded onto a HiLoad 16/60 Superdex75 prepgrade column equilibrated with 20 mM MES-NaOH, 120 mM NaCl, 5 mM DTT, pH 6.5 at a flow rate of 1 mL/min. Fractions containing the pure ^15^N,^13^C-labeled HHARI RING2 domain were pooled and concentrated. The resulting HHARI RING2 protein contained an additional “GS” at its N-termini as a result of its cloning and TEV cleavage. After purification, the concentration of human HHARI RING2 were determined using the better Bradford assay (Bio-Rad).

### NMR Spectroscopy

NMR samples for assignment and structure calculation of ^15^N,^13^C-labeled human HHARI RING2 were prepared in 20 mM MES-NaOH, 120 mM NaCl, 5 mM DTT, 10% D_2_O/90% H_2_O at pH 6.5. Samples were concentrated by ultrafiltration (Millipore, Mississauga, ON, Canada) to a final volume of 300 µL and transferred into a Shigemi tube. Imidazole (2 mM) was added to the sample as a pH indicator [20] to ensure that the pH of the sample did not change during data acquisition.

All NMR data were collected at 25°C using a Varian Inova 600 MHz NMR spectrometer equipped with a triple resonance probe and z-field gradients. Backbone and side chain assignments for HHARI RING2 were determined from the following experiments collected using the standard pulse sequences from the Varian Biopack: ^1^H-^15^N HSQC [21], aliphatic and aromatic ^1^H-^13^C HSQC [21,22], HNCO, HNCA, HNCACB [23], CBCA(CO)NH [24], and aliphatic and aromatic HCCH-TOCSY [25]. ^15^N-NOESY-HSQC experiments were collected with mixing times of 150 ms. ^13^C-NOESY-HSQC aliphatic and aromatic experiments were collected in 100% D_2_O using mixing times of 100 ms. Data were processed using NMRPipe and NMRDraw [26] and analyzed using NMRViewJ [27]. 2,2-dimethyl-2-silapentane-5-sulfonate salt (DSS) was used as the internal standard and referenced at 0 ppm, while ^13^C and ^15^N chemical shifts were indirectly referenced to DSS [28]. The NMR assignments for HHARI RING2 domain have been deposited to the Biological Magnetic Resonance Databank (www.bmrb.wisc.edu) under accession code 19315.

### Structure calculations and refinement

Structures for HHARI RING2 were calculated from a combination of manual and automatic NOE assignments using the program CYANA [29]. The standard CYANA protocol for automated structures was used with default settings involving eight cycles of structure generation and refinement (100 structures/round). Zn^2+^-coordinating cysteine residues were identified using Cα and Cβ chemical shifts [30] and the tautomeric state of histidine residues was determined from Cδ chemical shifts [31]. To not bias the fold of the domain, initial calculations were performed without any Zn^2+^-ion restraints. Once the fold was observed using only NOE distance restraints, zinc atoms were then added using virtual linkers and restraints between atom pairs (Zn–Sγ, Sγ–Cβ, His Nε2–Sγ and Sγ–Sγ) were imposed to maintain proper tetrahedral geometry around the zinc ion [32]. The final 50 calculated structures were water refined using a modified force field in Xplor-NIH [33,34] as previously described [11]. The 20 structures with the lowest NOE energies were chosen as representative of the calculation and were analyzed using Procheck [35] and MolProbity 4 [36] online software. The structures had a Molprobity score of 2.8 and clash score of 14.1. The atomic coordinates and structural restraints for HHARI RING2 have been deposited in the RCSB Protein Data Bank (www.rcsb.org) under accession code 2M9Y.

## Results and Discussion

### Structure of the HHARI RING2 Domain

**Figure 1:**
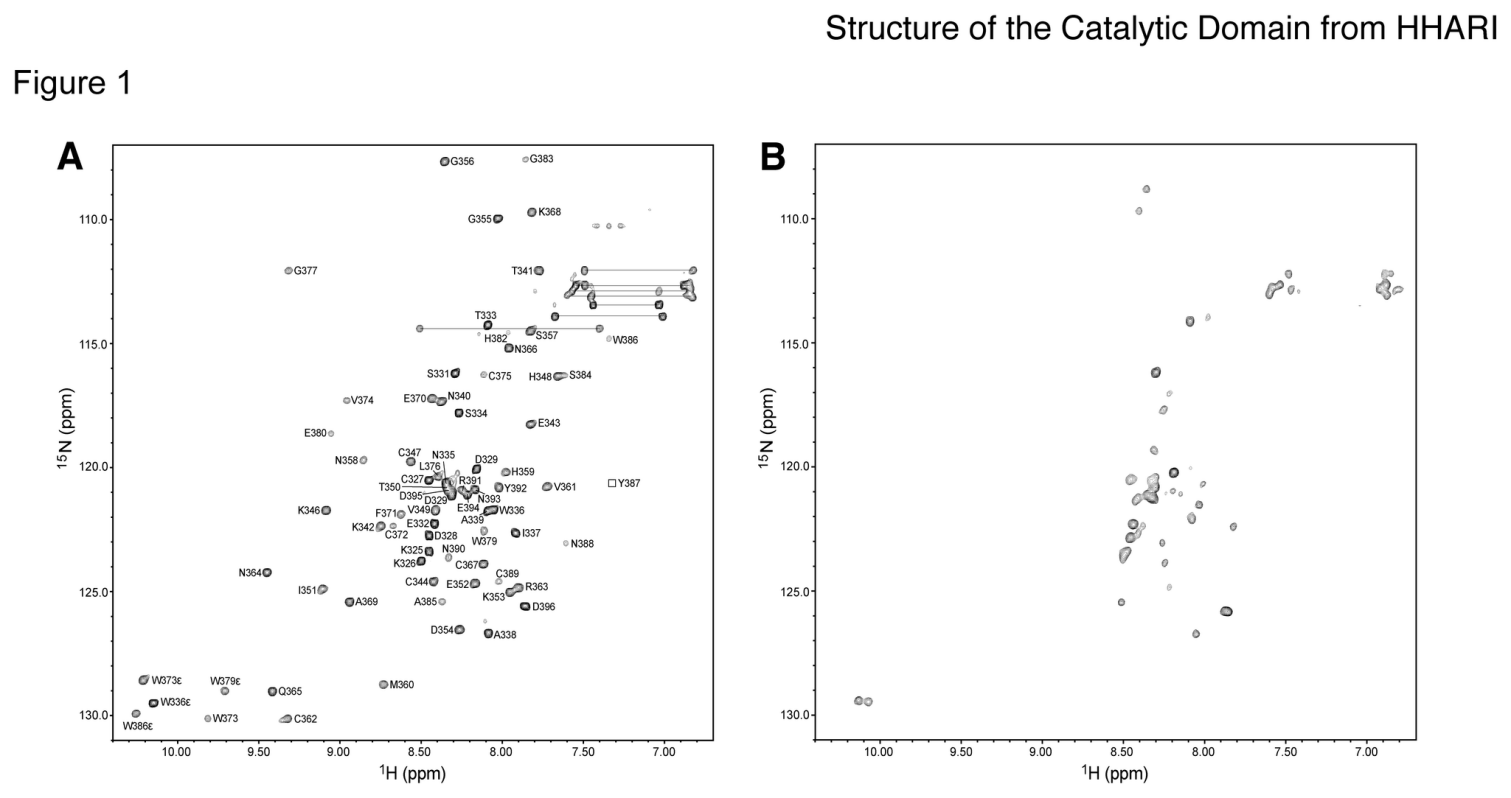
The HHARI RING2 domain structure is maintained by zinc binding. (**A**) Assigned 600 MHz ^1^H-^15^N HSQC spectrum of ^15^N,^13^C-labeled HHARI RING2 (C357S) domain (20 mM MES, 120 mM NaCl, 5 mM DTT, 10% D_2_O/90% H_2_O, pH 6.5) labeled using the one-letter amino acid code and residue number according to the human HHARI sequence. (**B**) ^1^H-^15^N HSQC spectrum of the HHARI RING2 (C357S) domain (20 mM MES, 120 mM NaCl, 5 mM DTT, 10% D_2_O/90% H_2_O, pH 6.5) in the presence of 4 mM EDTA. The collapsed amide peaks in the ^1^H dimension represent the unfolding of the protein due to the chelation of the structural Zn^2+^-ions.

Initial characterization of the human HHARI RING2 domain (residues K325-D396) used a C357S substitution to limit protein oxidation. The C357S substituted HHARI has been shown to successfully capture the C-terminus of ubiquitin and form an ester complex [10]. Examination of the HHARI RING2 domain by ^1^H-^15^N HSQC spectroscopy (Figure 1) showed dispersed amide resonances indicative of a well-folded protein. The addition of EDTA to the sample resulted in a collapse of the signals concurrent with significant changes in their intensity indicative of protein unfolding. For example, HHARI RING2 contains eight cysteine residues and four tryptophan residues that are found at well-separated positions in the native spectrum but are poorly resolved in the presence of EDTA. Since mass spectrometry experiments have shown that the HHARI RING2 domain coordinates two zinc ions [11], this indicates that zinc ion coordination is required for the correct folding of the HHARI RING2 domain.

**Figure 2:**
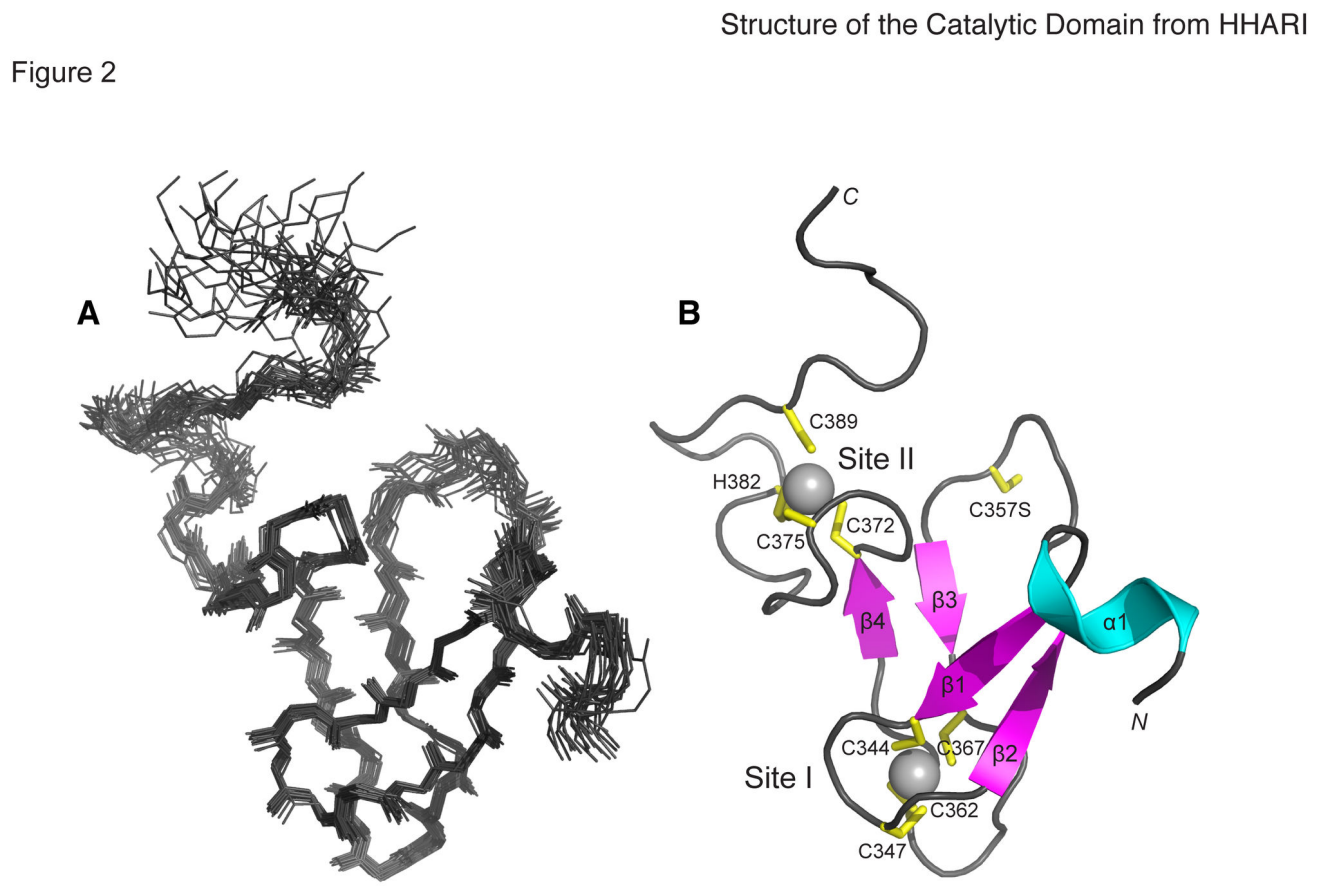
Solution Structure of the HHARI RING2 domain. (**A**) Superposition of the 20 lowest energy solution structures of the HHARI RING2 domain (residues 325-396; backbone RMSD 0.6 ± 0.2 Å). (**B**) Ribbon structure of the HHARI RING2 domain showing helix α1 (N335-A339) and β-strands β1 (T341-C344), β2 (V349-E352), β3 (H359-361), and β4 (E370-C372). Zinc is represented as silver spheres. Side chains of residues involved in Zn^2+^-coordination and the catalytic cysteine substituted with serine (C357S) are labeled and shown in yellow.

The solution structure of the HHARI RING2 domain was determined using a combination of NMR spectroscopy and structure calculations. In all, >95% of backbone and side chain assignments were determined for HHARI RING2 (Table 1) using standard triple-resonance NMR experiments. The structure determination used ^15^N and ^13^C-edited NOESY experiments that provided about 1500 non-redundant distance restraints as input for calculations (Table 1). The high number of distance measurements (~20 per residue) allowed structures to be calculated without accessory angular restraints. Initial structures were calculated in the absence of any Zn^2+^-ion restraints so as to not bias the fold of the domain. However, the identities of Zn^2+^-coordinating residues were obvious from chemical shift analysis of cysteine [30] and histidine [31] residues. The resulting family of 20 structures (Figure 2A) demonstrated that residues N335-C389 of HHARI RING2 form a well-structured domain (RMSD 0.65 Å) with a single turn α-helix (α1, N335-A339) followed by four well-defined βstrands (β1, T341-C344; β2, V349-E352; β3, H359-V361; and β4, E370-C372) (Figure 2B). The *N*- (K325-S334) and C-termini (R391-D396) of HHARI RING2 are disordered as supported by their chemical shift data and lack of long-range NOEs. Two Zn^2+^-binding sites are clearly present involving four cysteine residues found in the loops between β1-β2 and β3-β4 (Site I; C344, C347, C362, C367) and three cysteines and a histidine in the extended loop after β4 (Site II; C372, C375, H382, C389). This linear Zn^2+^-binding coordination is similar to that observed for the solution structure of the RING2 domain from the RBR E3 ligase parkin [11].

**Table 1:** Structural Statistics for 20 lowest energy structures of the HHARI RING2 Domain.

**Completeness of Resonance Assignments**	
Backbone (N, CA)	(141/145) -97.2%
Sidechain (C,H)	(522/540) -95.6%
HN	(68/71) -95.8%
HA	(76/79) -96.1%
HB	(122/122) -100%
**NMR distance and dihedral constraints**	
Distance constraints	
Total	1483
Intra-residue	364
Inter-residue	
Sequential (|*i* -*j*| = 1)	444
Medium-range (1<|*i* -*j*| < 5)	194
Long-range (|*i* -*j*| ≥ 5)	481
Intermolecular	0
Zinc coordination restraints	24
**Structure statistics**1	
Violations	
Distance constraints (> 0.1 Å)	0
Deviations from idealized geometry3	
Bond lengths (Å)	0.006
Bond angles (^°^)	0.56
Impropers (^°^)	0.74
Ramachandran Statistics2	
Most favored	70.0%
Additionally favored	29.2%
Generously favored	0.7%
Disallowed	0.1%
RMSD to Mean Structure (Å)3	
Backbone	0.6 ± 0.2
Heavy	0.8 ± 0.2

^1^ Using all residues, as reported by Xplor-NIH

^2^ As reported by Procheck

^3^ Using residues W336-C389 (inclusive)

Our structure of the HHARI RING2 domain does not resemble a previously determined structure [37] where only a single Zn^2+^ ion was coordinated (Site I) and the C-terminus was largely unstructured. This is likely due to our NMR assignment of resonances for aromatic residues, and resulting NOEs, which constitute a large portion of the C-terminus of HHARI RING2 (*vide infra*). This is manifested in differences in the second Zn^2+^-binding site where the current structure shows ligation through C372, C375, H382, and C389 not observed in the previous work. It is also possible that the observed structural differences could result from different loadings of zinc into site II of the HHARI RING2 domain. This site has Cys_2_-His–Cys coordinating ligands shown to have about a three-fold lower zinc affinity than a Cys_4_ geometry (Site I) in zinc finger peptides [38]. A partial occupancy of zinc may have contributed to sample heterogeneity and resulted in incomplete backbone and aromatic assignments in earlier work. We also found that the ^1^H-^15^N HSQC spectrum of HHARI RING2 was markedly improved at the lower pH (pH 6.5) used in the current work compared to pH 8 where the previous work was completed [37].

The HHARI RING2 structure shows a single solvent-exposed cysteine (C357; substituted to a serine in our construct, C357S) in the loop between β2-β3 that is not involved in Zn^2+^-coordination. This residue has been shown to form a covalent thioester with ubiquitin transferred from the E2 enzyme UbcH7 [10]. The resulting RING2~ubiquitin species is a required intermediate for the ubiquitin chain-building process to occur by HHARI. In a C357S substituted protein, it is also possible to form a more stable ester with the C-terminal carboxylate of ubiquitin although ubiquitin chain formation occurs more poorly [10]. Similar observations have been made for the RBR E3 ligases parkin [11] and HOIP [8,9] that also possess a conserved cysteine residue in their sequences.

### Conserved Aromatic Residues Maintain the HHARI RING2 Domain Structure

**Figure 3:**
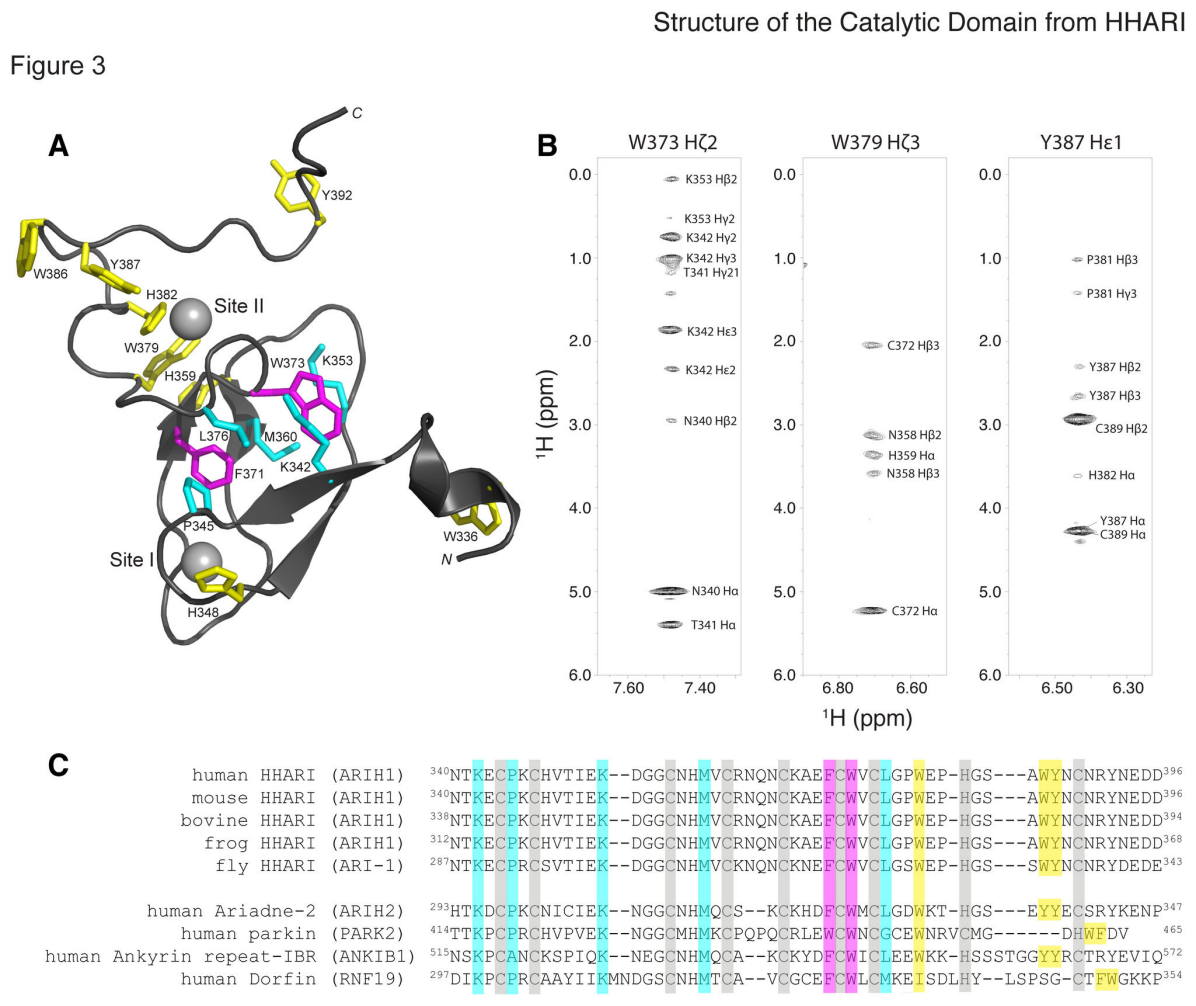
Conserved residues in the hydrophobic core of HHARI RING2 are conserved in all RBR RING2 domains. (**A**) The location of aromatic residues and key contacts in the core of the HHARI RING2 domain structure are shown for F371 and W373 (magenta), residues contacting F371 and W373 in the core (cyan), and all other aromatic residues (yellow). (**B**) Representative aromatic ^1^H-^1^H NOE strip plots for W373 Hζ2, W379 Hζ3, and Y387 Hε1 that make NOE contacts within the core, the β2–β3 loop containing the catalytic cysteine, and Zn^2+^-binding site II, respectively. (**C**) Sequence alignment of HHARI RING2 orthologs and representative RBR RING2 paralogs. Conserved aromatic residues in the core are colored as in panel A. Cysteine and histidine residues that coordinate Zn^2+^ (grey) are highlighted. Conserved residues corresponding to W379 and the tandem aromatic pair found towards the C-termini of the RBR sequences are highlighted in yellow (ie. W386 and Y387 in HHARI).

The structure of the HHARI RING2 domain shows several aromatic residues that are located near the core of the protein structure (Figure 3A). For example, W373 is found near Zn^2+^-binding site II that makes numerous NOE contacts with two conserved lysine residues K342 and K353 (Figure 3B). Other examples include W379 and Y387 located near the C-terminus of HHARI RING2 that make contacts within the loop containing the catalytic cysteine (C357S, H359) and Zn^2+^-binding site II (C372, C389), respectively. For the NMR structure determination, complete resonance assignments of all aromatic residues in HHARI RING2 were required in order to properly determine this conformation, a feature lacking in the earlier HHARI structure [37].

The RING2 domain of HHARI contains an unusually high percentage of aromatic residues (14%; 10 aromatic of 71 amino acids). We examined other RBR proteins to determine if a similar trend occurred for aromatic residues that were important for maintenance of the protein fold. Figure 3C shows that aromatic residues at F371, W373 and W379 are well conserved through different RBR proteins. For example, in both cases the tryptophan residue (W379 in HHARI, W453 in parkin) is juxtaposed to a conserved histidine (H359 HHARI, H433 in parkin) found in the β2-β3 loop that also carries the catalytic cysteine residue. This raises the possibility that W379 in HHARI RING2 may play an important role, whether directly or indirectly, in the ubiquitin transfer mechanism by the RBR E3 ligases.

Interestingly, several RBR E3 ligases also contain a tandem pair of aromatic residues near the C-terminal part of their Zn^2+^-binding site II (W386, Y387 in HHARI). This region is not obviously conserved through sequence alignment due to differential spacing of the last two Zn^2+^-coordinating residues and likely accounts for the inability of the C-terminus of HHARI to substitute for a similar region in parkin [39]. Nevertheless, the tandem aromatic residues appear to occupy similar spatial positions in the solution structures of the HHARI and parkin RING2 domains [11]. Based on the recent high resolution structure of parkin [40] the importance of one of the tandem aromatic residues has now been examined for this RBR E3 ligase. The structure shows the conserved phenylalanine -3 residues from the C-terminus of parkin (F463, equivalent to Y387 in HHARI) is involved in interactions with the RING0 domain. These interactions can be relieved when F463 is substituted with a tyrosine [40] leading to increased ubiquitination or covalent modification using a vinyl-sulfone probe. Likewise, the substitution of the final three residues of parkin containing F463 have been shown to be integral for proper folding and enzyme activity [39]. Future studies to examine the tandem aromatic residues near the C-terminus of the HHARI RING2 domain will be paramount to shed further light on their importance in RBR-dependent ubiquitin transfer.

### The RING2 Structure is a Conserved Feature of RBR E3 Ligase Proteins

**Figure 4:**
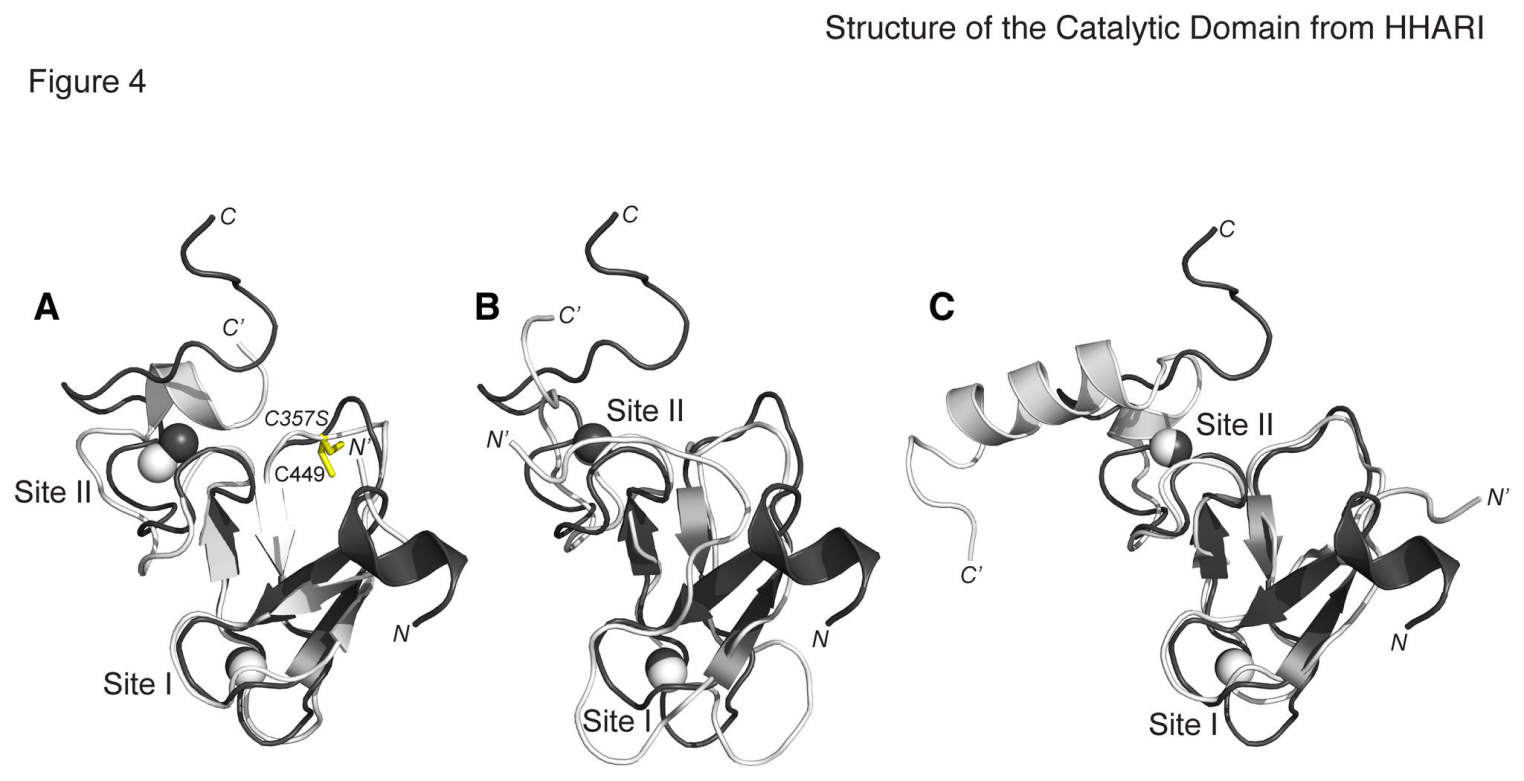
Superposition of HHARI RING2 domain with other RBR domains. The solution structure of the HHARI RING2 domain is oriented as in Figure 2B (grey) with *N*- and C-termini labeled. Solution structures of (**A**) parkin RING2 domain (PDB accession code 2LWR), (**B**) parkin IBR domain (PDB 2JMO), and (**C**) HOIP IBR domain (PDB 2CT7) are shown in white with *N*- and C-termini labeled (*N*’, *C*’). The conserved cysteine found in both the HHARI and parkin RING2 domains (yellow, panel A), is noticeably absent in the IBR domains.

We compared the solution structure of the HHARI RING2 domain with the solution structures of the parkin RING2 domain [11], and IBR domains from parkin [32] and HOIP (PDB accession code 2CT7). As shown in Figure 4, all four structures have a similar fold when compared to the HHARI RING2 structure (backbone RMSD parkin RING2 = 0.84 Å, parkin IBR = 1.38 Å, and HOIP IBR = 1.15 Å). The location of the catalytic cysteine within the loop between β2 and β3 is nearly identical for HHARI and parkin RING2 domains (Figure 4A; C357 in HHARI; C449 in fly parkin). The cysteine residue is conserved in all RING2 domains and suggests that the catalytic mechanism employed by each RBR is likely similar. Interestingly, even though the IBR domains from parkin and HOIP show a similar fold, both domains are lacking this cysteine residue.

The comparison of the HHARI RING2 domain structure with other IBR and RING2 domains indicates the most common features are the β-sheet structure near the center of the domains and the location of the two Zn^2+^ ions. There is variability of the spacing between ligating residues in both metal-ion sites leading to longer loops between the first and third pairs of ligands in site I (for example, the HHARI RING2 compared with the parkin IBR structure, Figure 4B). In addition the tandem pair of aromatic residues found near the C-terminus of the HHARI RING2 domain is absent from the IBR structures.

### The HHARI RING2 Structure shows a Glimpse of HECT Domain Structure

A current proposal is that HHARI and its RBR paralogs carry out the ubiquitination of their substrates using a hybrid mechanism combining aspects from both the RING and HECT E3 ligase families [8–10]. This is supported by biochemical assays that show a conserved cysteine found in all RBR RING2 domains can form a thioester (or non-reducible ester when substituted with serine) with ubiquitin adducts suggesting that the RING2 is the catalytic moiety in RBR proteins [8–10].

**Figure 5:**
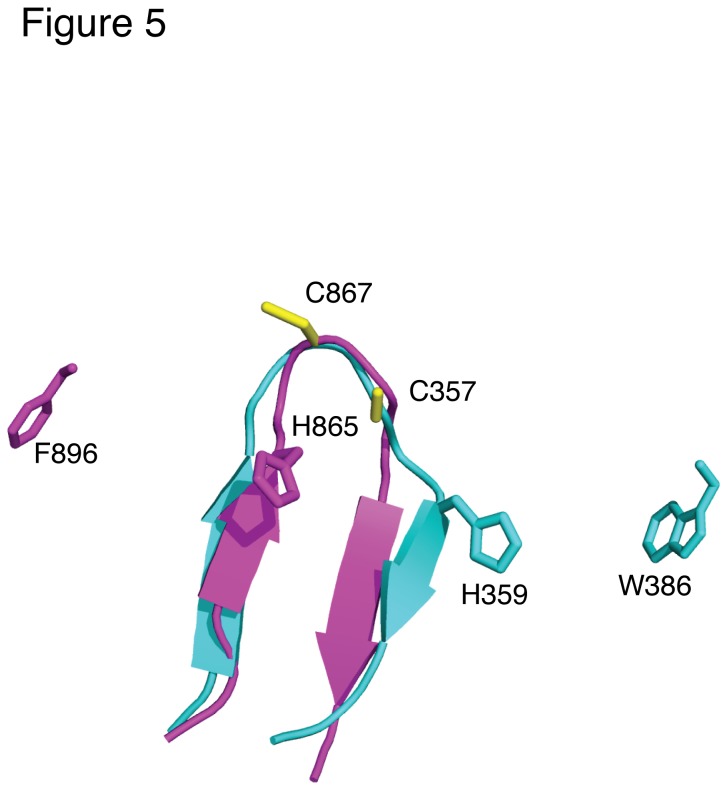
Structural similarities between catalytic regions of the HHARI RING2 domain and the HECT C-terminal lobe. Superposition of the HHARI RING2 domain (residues I353-C362, cyan) with the NEDD4 HECT domain (PDB 4BBN; residues P861-D871, magenta). The catalytic cysteine residues are each located near the center of the loop connecting an antiparallel β-sheet (β2-β3 in HHARI). Histidine residues shown to be important in ubiquitination (H359 for HHARI, H865 for NEDD4) are located two residues away from the catalytic cysteine. In HHARI, an aromatic residue (W386) conserved as part of a tandem aromatic pair in RBR E3 ligases is indicated. A similar aromatic residue (F896) in NEDD4 is required for ubiquitin transfer to a substrate.

The hybrid mechanism would suggest that some structural features of a HECT E3 ligase might exist within the RING2 domain of HHARI or other RBR E3 ligases. In order to examine this, we compared the catalytic region of HHARI with that of the HECT E3 ligase NEDD4 [41]. As shown in Figure 5, some similarity exists within the catalytic portions of both proteins, although the two proteins appear to be mirror images of each other. In both cases, a catalytic cysteine (C357 in HHARI; C867 in NEDD4) lies within a loop between two antiparallel β-strands. Further, both E3 ligases feature a histidine residue located two residues prior to the cysteine in the case of NEDD4 (H865) or afterwards for HHARI (H359) and parkin (H433). Substitution of H920 in NEDD4L affects the transthiolation reaction from the E2 enzyme to the catalytic cysteine [42] while substitution of H433 in parkin nullifies the polyubiquitin reaction [11] and severely reduces reactivity to a ubiquitin vinyl-sulfone probe [40]. Further, in NEDD4 it has been shown that substitution of a single conserved phenylalanine (F896) located about 30 residues past the catalytic cysteine blocks ubiquitin transfer to the substrate [43]. This residue has been suggested to orient the thioester-bound ubiquitin or move closer to the catalytic site during ubiquitin transfer [41,43]. It is interesting that W386 in the HHARI domain (W462 in parkin) within the conserved tandem aromatic pair of the RING2 domain might fulfill such a role in the HECT/RING hybrid mechanism since this residue is located about 30 residues C-terminal to the catalytic cysteine and occupies a near mirror image position compared to F896 in NEDD4. These intriguing observations show there are some structural similarities between HECT E3 ligases and the RING2 domains from HHARI and parkin that are consistent with a HECT/RING hybrid mechanism. These results provide a framework for future experiments to further unravel the hybrid mechanism used by the RBR E3 ligases.
